# A solution for factorial validity testing of three-item scales: An example of tau-equivalent strict measurement invariance of three-item loneliness scale

**DOI:** 10.1007/s12144-021-01554-5

**Published:** 2021-03-07

**Authors:** Stanisław K. Czerwiński, Paweł Andrzej Atroszko

**Affiliations:** Institute of Psychology, University of Gdańsk, Bażyńskiego 4, 80-309 Gdańsk, Poland

**Keywords:** Loneliness, Measurement invariance, Quality of life, Scale, Tau-equivalence, Validity

## Abstract

Ultra-short scales are increasingly popular in surveys. Congeneric model fit of a three-item scale cannot be tested with Confirmatory Factor Analysis (CFA) without additional assumptions because the number of degrees of freedom is equal to zero. A more rigorous tau-equivalent model, assuming equality of factor loadings can be tested instead. The objective of this study was to demonstrate this approach with an example of the psychometric study of the Polish version of the Three-Item Loneliness Scale (TILS), and to discuss the arising problems and possible solutions. There seems to be a high need for such analysis because currently, some properties of CFA make it an approach still predominant over Item Response Theory (IRT) models in the quality of life research. A sample of 3510 students completed TILS together with the questionnaires measuring a variety of indicators of well-being. The results provided evidence for a good fit of a tau-equivalent model. Furthermore, multi-group CFAs provided support for strict measurement invariance of this model. To the Authors’ knowledge, it is the first practical application of a tau-equivalent model to testing the factorial validity of an ultra-short scale and probably the first empirical case of tau-equivalent measurement invariance in psychological literature in general. TILS showed good criterion validity and satisfactory reliability. Unidimensionality of three-item scales can be examined with a tau-equivalent model that has some favorable psychometric properties. However, it might be exceedingly restrictive in certain practical cases. When developing a new short scale, it is recommended to maintain at least four items.

## Introduction

### Loneliness

Loneliness is defined by de Jong Gierveld as ‘a situation experienced by a participant as one where there is an unpleasant or unacceptable lack of (quality of) certain social relationships. The extent to which the situation is experienced as serious depends upon the participant’s perception of his or her ability to realize new relationships, or to improve existing ones.’ (de Jong Gierveld, 1989). It is important to emphasize that loneliness is a subjective feeling and to distinguish it from social isolation, as they are two separate constructs (Coyle & Dugan, 2012; Perissinotto & Covinsky, 2014), for one can be in the company of others and still feel alone or live in seclusion and have no negative feelings about it whatsoever.

A large number of studies concerning loneliness focus on the elderly population (Holt-Lunstad, Smith, Baker, Harris, & Stephenson, 2015) whilst neglecting younger samples, even though the relationship between age and loneliness has been found to be U-shaped (Pinquart & Sorensen, 2001). Loneliness is not a problem specific only to the elderly. Studies show that younger generations are showing less concern for other people and gravitate away from civic orientation (Twenge, Campbell, & Freeman, 2012), which in turn could explain their increase in loneliness (Cigna U.S. Loneliness Index, 2018; Twenge, Spitzberg, & Campbell, 2019). With social support being one of the fundamental factors in human well-being (Cohen & Wills, 1985), it is not surprising that each successive generation shows more and more signs of mental problems (Twenge et al., 2010). Poland could be exceptionally vulnerable to this effect. For example, it is one of the few countries in Europe to have increasing suicide rates (Höfer, Rockett, Värnik, Etzersdorfer, & Kapusta, 2012). The rapid social change in post-communist countries is among the postulated causes for a decrease in well-being (Domagała-Krecioch & Majerek, 2014; Höfer et al., 2012). Poland as a country that transited to the market economy and is still in the process of adapting the Western “way of life” is a compelling case for the process of increasing alienation among youth (Zoutewelle-Terovan & Liefbroer, 2017). The existing data seems to support the notion that dynamic economic growth comes with a price. For example, compulsive overworking prevalence evaluated with the same measure and cut-off score seems to be 2 to 3 times higher in Poland than in countries with established and stable economies such as Denmark and Norway (Andreassen, Nielsen, Pallesen, & Gjerstad, 2019; Atroszko, Pallesen, Griffiths, & Andreassen, 2017; Lichtenstein, Malkenes, Sibbersen, & Hinze, 2019), and this trend is already visible among undergraduate students (Lawendowski, Bereznowski, Wróbel, Kierzkowski, & Atroszko, 2019). Having that in mind, further investigation of loneliness in young adults, especially in rapidly economically developing countries, is of great importance. Our study focused on the undergraduate students because they comprise almost half of the population age 19–24 in Poland (Główny Urząd Statystyczny, 2019) and previous studies showed that student populations are highly vulnerable to loneliness (American College Health Association, 2016). Polish studies showed high prevalence of depression and considerable hopelessness among university students (Czerwiński, Mackiewicz, Mytlewska, & Atroszko, 2020; Koryczan, Piotrowski, Roj, Czerwiński, & Atroszko, 2020).

The previous studies showed significant relationships of loneliness with a wide range of well-being indicators, such as health (Swami et al., 2007; Tobiasz-Adamczyk et al., 2017), sleep quality (Matthews et al., 2017; Yu, Steptoe, Niu, Ku, & Chen, 2017), self-esteem (Heinrich & Gullone, 2006; Vanhalst, Luyckx, Scholte, Engels, & Goossens, 2013), satisfaction with personal relationships (Mellor, Stokes, Firth, Hayashi, & Cummins, 2008), social support (Segrin & Passalacqua, 2010; Tobiasz-Adamczyk et al., 2017), general quality of life (Fanakidou et al., 2017), satisfaction with life (Buelga, Musitu, Murgui, & Pons, 2008; Huo & Kong, 2014; Liu & Guo, 2008), stress (Hughes, Waite, Hawkley, & Cacioppo, 2004; Lee & Goldstein, 2015), anxiety (Fanakidou et al., 2017; Moore & Schultz, 1983), and depression (Cacioppo, Hughes, Waite, Hawkley, & Thisted, 2006; Erzen & Çikrikci, 2018).

### Issues in Factorial Validity Testing of Three-Item Scales

Ultra-short scales are becoming increasingly popular in educational and psychological research due to the convenience of application, often satisfactory psychometric properties and reduction of bias introduced by the excessive burden on participants with long questionnaires. They are often used, for example, in the quality of life research (Cheung & Lucas, 2014), health psychology, including clinical settings (Krebs et al., 2009), and epidemiology (Beutel et al., 2017) due to the convenience of their use. Since loneliness is a variable strictly related to the well-being (Erzen & Çikrikci, 2018; Rico-Uribe et al., 2016; Valtorta, Kanaan, Gilbody, Ronzi, & Hanratty, 2016; Vanderweele, Hawkley, & Cacioppo, 2012), we will discuss the psychometric issues related to the ultra-brief scales within the context of quality of life research and health psychology. Even though in the case of individual diagnosis a smaller pool of items comes with a significant loss in precision, when conducting a large scale surveys focused on the relationships between numerous variables and controlling for a wide range of covariates, more concise instruments usually perform almost as good as their lengthier alternatives (Gogol et al., 2014; Kemper, Trapp, Kathmann, Samuel, & Ziegler, 2018; Rammstedt & Beierlein, 2014), including having good predictive validity (Bergkvist & Rossiter, 2007). In such case, short, valid and reliable tools are invaluable. In consequence, a proper analytical approach to substantiate their adequate psychometric properties is crucial and poses specific challenges. For example, the reliability of single-item measures cannot be substantiated with internal consistency coefficients, and may require test-retest coefficients such as Intraclass Correlation Coefficient (ICC). It should be emphasized that the focus of this paper is on the specific circumstances in which the already existing ultra-short scale is being validated in a new sample, such as in different culture or different demographic. The measure is not used for precise diagnosis of individuals but to investigate the relationships with other variables or as a means to control for a confounding variable, for example, as in Health and Retirement Study (Chen & Feeley, 2014). Furthermore, such a measure can be particularly valuable if it was used extensively in previous studies and showed good psychometric properties, such as in the case of the Three-Item Loneliness Scale (TILS). This is of the highest importance from the perspective of the direct comparisons of results and emphasis laid on the reproducibility and replicability of effects (Open Science Collaboration, 2015; Pashler & Wagenmakers, 2012; Patil, Peng, & Leek, 2016; Plesser, 2018). In this context, a specific class of ultra-short measures is three-item scales which are not infrequently used in the quality of life research (Cuijpers, Smits, Donker, Ten Have, & de Graaf, 2009; Jenkins, Stanton, Niemcryk, & Rose, 1988; Kelly, 2004; Krebs et al., 2009; Leon, Olfson, Portera, Farber, & Sheehan, 1997).

The standard psychometric approach is the latent variable model (Borsboom, Mellenbergh, & Van Heerden, 2003; Borsboom, Molenaar, & Wright, 2015; Muthén, 1984; Smith, 2000). It is a statistical model that relates a set of observable/manifest variables to one or more latent variables. In such a model, loneliness is assumed to be a latent continuous variable that is measured by either continuous or categorical manifest variables. One of the first steps in the validation process aimed at substantiating construct validity is demonstrating that the items measure the same construct. This is most often performed with factor analysis or methods more suitable for nonlinear data often used for Rasch models and Item Response Theory (IRT) models, such as nonlinear factor analysis or multidimensional scaling (De Ayala & Hertzog, 1991), or item fit approach (Tay & Drasgow, 2012), and other methods with specific approach depending on the manifest variable measurement scale and linearity/nonlinearity of the relationship between item score and latent trait (Smith, 2000; Stochl, Jones, & Croudace, 2012). An overview of the proposed procedures for dimensionality testing of a set of item responses can be found elsewhere (De Champlain & Gessaroli, 1998). In some cases, including ordered categorical data, IRT models are equivalent to factor analysis (Bartholomew, 1987; Birnbaum, 1968; Samejima, 1970; Takane & De Leeuw, 1987), and both models allow for measurement invariance testing (Kim & Yoon, 2011; Muthén & Asparouhov, 2014). The choice of a specific psychometric approach is determined by multiple factors (Petrillo, Cano, McLeod, & Coon, 2015), and there are still no explicit guidelines of which solution is preferable in all possible circumstances (Coulacoglou & Saklofske, 2017). The detailed discussion of the technicalities behind particular approaches as well as philosophical underpinnings behind the latent variable model exceeds the scope of this paper and can be found elsewhere (Borsboom et al., 2003; Coulacoglou & Saklofske, 2017). While acknowledging the ontological doubts regarding the status of latent variables, this paper focuses on the solutions related to confirmatory factor analytic (CFA) approach (sometimes called “restricted factor analysis” (McDonald, 2013)) which is one of the most widely used statistical approaches in the quality of life research, and in social and behavioral sciences in general (Depaoli, Tiemensma, & Felt, 2018).

The advantages of this approach include its congruence with modern scientific standards based on hypothesis testing and confirmatory approach (Gorsuch, 1983; Wagenmakers, Wetzels, Borsboom, van der Maas, & Kievit, 2012), relative ease of practical application for quality of life researchers non-specializing in advanced statistics, and suitability for particular practical aims such as using a measure to enable investigating the relationships between variables and controlling for covariates in large scale surveys. Studies show that for specific purposes such as measurement invariance testing, CFA results are to a large extent similar to Rasch measurement and can be treated as a good approximation of a more comprehensive approach (Salzberger & Sinkovics, 2006). These properties of CFA make it an approach overwhelmingly predominant over IRT models in the quality of life research (Depaoli et al., 2018). Possible explanations for why IRT approaches are rarely used in health-related studies include them being perceived as challenging to implement and interpret or requiring large sample sizes that are oftentimes difficult or impossible to obtain, especially in clinical populations (Salzberger & Sinkovics, 2006).

Within the CFA approach, there are different models used for testing the structure of a measure depending on the assumptions underlying the measurement. The most restrictive *parallel model* requires that all items must measure the same latent variable, on the same scale, with the same degree of precision, and with the same amount of error (Raykov, 1997a, 1997b). The *tau-equivalent model* allows individual item error variances to differ from one another. The *essentially tau-equivalent model* further allows for different degrees of precision of measurement. The least restrictive and the most general *congeneric model* assumes that each item measures the same latent variable, with possibly different scales, different degrees of precision, and with different amounts of error. Even though commonly used Cronbach’s alpha coefficient requires at least a tau equivalent model, most of the studies use the congeneric model due to its least restrictive nature, and consequently report biased estimates of internal consistency with Cronbach’s alpha. For more up to date and approachable description of the models, please see for example paper by Graham (2006).

In the case of single-factor three-item scales, the congeneric measurement model is just-identified, has zero degrees of freedom, and thus its fit to the data cannot be meaningfully tested. This problem can be solved by assuming a more rigorous tau-equivalent model which constrains all loadings to be held equal (Graham, 2006). Thus, it yields a non-zero number of degrees of freedom and makes it possible to test the model’s goodness of fit. With three items, high and equal loadings provide favorable psychometric properties in terms of factorial validity and reliability, and therefore improve other types of validity such as criterion validity (especially when sums of items instead of latent factors are used in the subsequent analyses). To the Authors’ knowledge, tau-equivalence has never been tested before in a practical setting due to strict and often empirically unfeasible assumptions that it takes. Furthermore, testing measurement invariance of a tau-equivalent model in most cases would yield unsatisfactory results due to its extremely rigorous assumptions. Therefore, while it seems empirically feasible to develop ultra-short tau-equivalent scales, the subsequent invariance testing of such scales across a variety of groups could bring more problems than benefits.

There are few other possible solutions to the problem of the factorial validity of three-item scales, such as using Rasch or IRT model, analyzing a model with more items and assuming unidimensionality of the shorter version if it was confirmed for the longer version, and analyzing scale together with other scales in a multifactor model. However, when taking into account specific circumstances of interest, i.e., validating already existing three-item measure used for investigating associations with other variables, these solutions have some disadvantages in comparison to testing a tau-equivalent model.

The first alternative could be a polytomous Rasch model or a more flexible graded response model (Baker, Rounds, & Zevon, 2000; Samejima, 1970; Tuerlinckx & Wang, 2004). These approaches are commonly used to analyze the performance of particular items with multiple category responses without guessing. As it was already mentioned, these approaches were shown to be equivalent to CFA; however, they provide more sophisticated information (Jöreskog & Moustaki, 2001; Kamata & Bauer, 2008; Takane & De Leeuw, 1987). While arguably they could be used to investigate the psychometric performance of a three-item scale, they require more expertise with psychometrics, larger sample sizes, and preferably more items (De Champlain & Gessaroli, 1998). These analyses represent a feasible, challenging, perhaps fascinating but conceivably disproportionately overcomplicated approach in the discussed context. Studies show that for specific purposes such as measurement invariance testing (Kim & Yoon, 2011; Meade & Lautenschlager, 2004; Muthén & Asparouhov, 2014; Samejima, 1970; Tay, Meade, & Cao, 2015), CFA results are to a large extent similar to Rasch measurement and can be treated as a good approximation of a more comprehensive approach (Salzberger & Sinkovics, 2006).

A different possible solution to the problem of three-item scales having zero degrees of freedom could be testing them alongside another measure in a multi-factor solution. This, however, brings a few problems. Firstly, the other scale(s) used in such a model should be very carefully chosen. It should be preferably more than three-item so that its fit could be independently examined. Secondly, it should measure a somewhat dissimilar construct in order to minimize any overlapping, which could affect the fit of the multifactor model. However, had such a model failed to show satisfactory fit, sometimes it could be challenging to identify the exact source of problems as in CFA models, problems with fit tend to propagate throughout the model. For example, residuals of items pertaining to one scale could have substantial covariance with residuals of items of another scale. This would require allowing for correlated error terms in order to obtain acceptable fit, which equals to imposing arbitrary ad hoc assumptions on the model. Additionally, subsequent studies would have to use the same scales to allow for comparing the results, and sampling variability could influence the results on any of the measures making it virtually impossible to conduct meaningfully such comparisons.

Another alternative could be the analysis of a congeneric model with more items, e.g., a full 20-item Revised UCLA Loneliness Scale (R-UCLA; Russell, Peplau, & Cutrona, 1980). If the unidimensionality of the full scale showed a good fit to the data, then it could be assumed that the shorter version of the scale also shows a good fit for a single factor solution. However, this approach still does not provide an exact measure of fit for the short three-item version of the scale. Furthermore, if only the three-item version is used in the subsequent studies, the fit of the data in a new sample would be unknown. This approach is related to another issue, i.e., potential multidimensionality of loneliness. One could argue that if a longer version of the scale is multidimensional and each item of the shorter version is taken from different factors, then the shorter measure is not unidimensional. However, there are two issues that need to be taken into account in this situation.

Firstly, even multidimensional measures of loneliness need to assume one general construct of loneliness. Otherwise, there would be three different but interrelated constructs. That would mean that there is no such phenomenon as loneliness but multiplicity of different types of loneliness, which poses practical and ontological problems. Therefore, a multidimensional measure of loneliness would have to assume a single general factor with several first-order factors measuring specific components of loneliness (Salzberger & Sinkovics, 2006). The correlated factors of loneliness most often stem from the inadequate analytical approach such as exploratory factor analysis (Mahon & Yarcheski, 1990; Mahon, Yarcheski, & Yarcheski, 1995; Wilson, Cutts, Lees, Mapungwana, & Maunganidze, 1992) or principal component analysis (Joiner, Catanzaro, Rudd, & Rajab, 1999) without cross-validation with CFA or CFA without testing model with higher-order factor (Goossens et al., 2009; Goossens & Beyers, 2002; Maes, Klimstra, Noortgate, & Goossens, 2014; Penning, Liu, & Chou, 2013). This is incongruent with the assumptions of hypothesis testing underlying the modern scientific method (Wagenmakers et al., 2012). Very few studies on loneliness investigate hierarchical structures, and when they do, it still does not conform to the strict requirements of confirmatory approach (Joiner et al., 1999). What follows from the assumption of one multidimensional construct of loneliness is that even three items, each measuring a different component of loneliness, still measure one general construct of loneliness. However, whether these items would show unidimensionality or not is another fascinating and complex psychometric problem which is yet to be systematically approached in simulation studies.

Secondly, a more technical problem with studies yielding multidimensional models of loneliness is that, to some extent, the multidimensionality seems to be an artifact of the item wording. TILS is a shorter version of R-UCLA (Hughes et al., 2004), one of the most commonly used measures of loneliness. R-UCLA originally consisted of 20 items and was assumed to have a single factor solution (Russell et al., 1980). Later, some studies provided evidence that two or three-factor solutions have a better fit for the instrument, with the most popular one being a three-factor model of “intimate others”, “social others” and “belonging and affiliation” (Austin, 1983; Kwiatkowska, Rogoza, & Kwiatkowska, 2017; McWhirter, 1990). However, it was argued that the multifactor solutions are, to some extent, an artifact of reversed item wording, and with the factors being very strongly correlated, the scale is measuring a singular construct of loneliness (Hartshorne, 1993; Russell, 1996). It is worth noting that in the case of another widely used measure of loneliness, De Jong Gierveld Loneliness Scale (de Jong Gierveld & Kamphuls, 1985), commonly used as a two-dimensional instrument with one dimension being comprised of only positively worded items and another with only negatively worded items, a bifactor analyses provided evidence that all items were better represented by one general factor of loneliness (Grygiel, Humenny, & Rębisz, 2016; Grygiel, Humenny, Rebisz, Świtaj, & Sikorska, 2013). The factorial artifacts produced by reverse coding of items seem to be a common problem in the quality of life research, and more attention should be devoted to making researchers more aware of it since many of the most widely used tools face this problem (Røysamb & Strype, 2002; Salerno, Ingoglia, & Coco, 2017). More information on analytical approaches to bifactor models can be found elsewhere (Chen, West, & Sousa, 2006; Tay & Drasgow, 2012; Wang, Chen, & Jin, 2014). Regardless of dimensionality controversies surrounding R-UCLA, the three items forming TILS are all negatively worded and derive from the same factor.

In order to meaningfully compare results obtained in different groups, measurement invariance of a scale should be demonstrated. The most commonly used method to test factorial invariance of a measure is based in CFA (Marsh, 1987), which is to a large extent, equivalent to the methods used in Rasch and IRT models (Salzberger & Sinkovics, 2006). This allows testing the equivalence of a scale across multiple groups. A more detailed description of the meaning and statistics behind testing invariance can be found elsewhere (Putnick & Bornstein, 2016).

### Present Study

The present study examined the psychometric properties of the Polish version of TILS in the student sample, including tau-equivalent measurement invariance between genders. The scale showed both concurrent and discriminant validity in previous studies, and satisfactory reliability, however, its factorial validity has not been effectively tested before, as the researchers mostly focused on criterion validity, tested measurement invariance without testing the model fit on its own or used exploratory factor analysis (Hawkley, Duvoisin, Ackva, Murdoch, & Luhmann, 2015; Hughes et al., 2004; Matthews-Ewald & Zullig, 2013).

## Materials and Methods

### Participants

The sample consisted of 3510 students from nine Polish universities, of which 1970 (56.1%) were female, and 1506 (42.9%) were male (34 respondents did not specify their gender). Participants’ mean age was 20.92 years (*SD* = 2.65). Thirty eight participants took part in test-retest procedure with three-week interval between measurements, 31 females and 7 males, with mean age of 20.14 years (*SD* = 1.31). Convenience sampling was used; however, in principle, it was aimed at assuring diversity of students to some extent representing the population of undergraduate students in Poland. Therefore, the sample included most of the types of universities (e.g., technological, business schools, humanistic, sport academy), both public and private, variety of faculties and courses of study from each university, both full-time and part-time students, and students of all years of study.

### Measures

The measures used were chosen due to their brevity, convenience of application, good psychometric properties and a need to reduce bias due to long survey fulfillment time typical for lengthier tools. All these measures are or are based on a widely recognized and commonly applied questionnaires.

Three-Item Loneliness Scale (TILS) consists of three items: *“How often do you feel that you lack companionship?”, “How often do you feel left out?” and “How often do you feel isolated from others?”.* The response options for each item were *“hardly ever,” “some of the time” or “often.”* The previous studies suggest that it has good validity and reliability, and measurement invariance in different countries, however, inadequate psychometric models applied in those studies need to be taken into account (Hawkley et al., 2015; Matthews-Ewald & Zullig, 2013). In the present sample, the Cronbach’s alpha reliability coefficient was .80.

General health, sleep quality, global self-esteem, satisfaction with personal relationships, satisfaction with support received from friends, general quality of life, satisfaction with life and meaning in life were measured with single-item measures with nine-point response scales developed on the basis of items from WHOQOL-BREF scale (Skevington, Lotfy, & O'Connell, 2004). The scales showed good validity and reliability in previous research (Atroszko, Bagińska, Mokosińska, Sawicki, & Atroszko, 2015; Atroszko, Pianka, Raczyńska, & Atroszko, 2015; Atroszko, Sawicki, Mąkinia, & Atroszko, 2017; Atroszko, Sawicki, Sendal, & Atroszko, 2017). In the previous studies, the reported ICC for test-retest reliability were .72 for general health, .81 for sleep quality, .79 for global self-esteem, .80 for satisfaction with personal relationships, .64 for support received from friends, .86 for general quality of life, .88 for satisfaction with life and .86 for meaning in life.

Perceived stress was measured with the Perceived Stress Scale-4 (PSS-4; Cohen, Kamarck, & Mermelstein, 1983). The PSS-4 consists of 4 items, a 5-point Likert response format scale, rated from 0 – *“Never”* to 4 – *“Very often.”* The Polish version of the scale showed good validity and reliability in previous research (Atroszko, 2015). In the present sample, the Cronbach’s alpha reliability coefficient was .72.

Anxiety and depression were measured by the Hospital Anxiety and Depression Scale (HADS), which includes 14 items with a 4-point response format (Zigmond & Snaith, 1983). Seven items measure anxiety, and seven measure depression. The Polish version of the scale showed good validity and reliability in previous research (Czerwiński et al., 2020). In the present sample, the Cronbach’s alpha reliability coefficient was .74 for depression, and .85 for anxiety.

### Procedure

Convenience sampling was used. Those willing to participate (more than 95%) filled in ‘paper and pencil’ anonymous questionnaires during regular university classes. There were no specific inclusion and exclusion criteria. No monetary reward was provided as an incentive to complete the survey. Attaining formal and written informed consent was not regarded as necessary as voluntary completion of the questionnaires was regarded as providing consent. Data were gathered between 2013 and 2017 as a part of subsequent research projects concerning survey studies on behavioral addictions among undergraduate students in Poland. The invitation to the study stated that it concerns psychosocial functioning of students, including wide range of variables such as personality and well-being. The order of presentation of the measures was changed a few times during the course of the projects in order to minimize the potential bias related to questions’ order. Data was gathered both by the principal investigator and research assistants.

The scale was translated from English to Polish in a multi-step translation process conforming to the commonly used standards of psychometric instruments translation. The process included the following procedures: i) translation from English into Polish separately by one bilingual person and one psychologist fluent in English, ii) developing an agreement on the initial Polish version within a panel consisting of both translators and a psychometrician, iii) back translation by two different translators: a bilingual person and a psychologist fluent in English, iv) comparing back translation with the original version and with initial Polish translation within a panel consisting of all four translators and a psychometrician, and choosing item wording for the final Polish version, v) pre testing among a group of individuals (*n* = 15) for any problems with understanding the items and their intended meaning, and introducing any necessary corrections to items’ wording.

### Statistical Analyses

Missing data (less than 1.5%) were imputed using expectation maximization (EM) algorithm, which provides unbiased estimates of parameters and improves statistical power of analyses (Enders, 2001; Scheffer, 2002). Confirmatory factor analyses were performed using Mplus 6.11. Due to the strictly ordinal character of the response scale, the CFA models were tested using Weighted Least Square Mean and Variance Adjusted (WLSMV) estimator. Due to the number of items, the standard congeneric model had zero degrees of freedom and could not yield meaningful results on the model fit to data. A tau-equivalent model was tested instead, as it increases the number of degrees of freedom to two. Tau-equivalence means that all items in the model load unto one factor equally. The basic model was initially tested separately in two groups of females and males. The following measures were used to evaluate the fit of the model: χ2 divided by degrees of freedom (*χ2/df*), Comparative Fit Index (CFI), Tucker-Lewis Index (TLI), Root Mean Squared Error of Approximation (RMSEA). CFI and TLI values above .95 are indicative of good fit (Hu & Bentler, 1999), however values above .90 are considered as adequate (Kline, 2004). RMSEA scores of .08 and lower are acceptable (Brown & Cudeck, 1993). Measurement invariance between genders was assessed using multiple-group procedures in which sets of parameters were freed sequentially in a series of hierarchically nested models. These models were also tau-equivalent. Configural invariance means testing the equivalence of factor structure between groups in the first step. Metric and scalar invariance could not be tested due to the structure of the response scale (three items with a three-point response format). Metric invariance assumes the same factor loadings between the groups, and scalar invariance assumes the same thresholds for items between groups. However, for ordered ternary data, any set of parameters with configural invariance can be transformed to satisfy the invariance of thresholds, of loadings and intercepts, or of intercepts and unique variances. As a result, there is no degree of freedom to test these invariance conditions (Wu & Estabrook, 2016). In consequence, metric and scalar invariance were not tested as, in this case, they are equivalent to configural invariance. As a result, only residual invariance can be meaningfully compared to the previous step. Strict invariance is the most rigorous model which tests whether the construct, item loadings, item thresholds, and residual variances are all the same in both groups. ICC along with the 95% confidence interval (CI) was used as a measure of test-retest reliability. Means, standard deviations, percentages, skewness and kurtosis of scales, Cronbach’s α for reliability, ICC and Pearson correlation coefficients were calculated using IBM SPSS 25.

## Results

Descriptive data on TILS is presented in Table 1. An ICC for TILS was .84 (95% CI = .67–.92, *p* < .001). It indicates good test-retest reliability.

**Table 1:** Response rates of each of the TILS items

	Entire sample			Females			Males		
	hardly ever (1)	some of the time (2)	often (3)	hardly ever (1)	some of the time (2)	often (3)	hardly ever (1)	some of the time (2)	often (3)
How often do you feel that you lack companionship?	43.6%	46.1%	10.1%	42.7%	47.4%	9.8%	44.6%	44.4%	11.0%
How often do you feel left out?	56.9%	31.5%	11.6%	55.3%	33.6%	11.2%	58.7%	29.0%	12.1%
How often do you feel isolated from others?	60.0%	29.1%	10.8%	60.4%	28.8%	10.7%	59.6%	29.5%	10.9%

In the female group a tau-equivalent one-factor model showed following fit indices: χ^2^/*df* = 10.44, CFI = .997, TLI = .995, RMSEA = .070 (90% CI = .045–.099), with each item loading of .86. In the male group, model fit indices were: χ^2^/*df* = 8.61, CFI = .996, TLI = .993, RMSEA = .071 (90% CI = .043–.104), and with each item loading of .84. Table 2 shows the model fit for each successively stringent test of invariance. The indicators of a model fit showed acceptable strict measurement invariance of a tau-equivalent model.

**Table 2:** Measurement invariance between genders

Model	*χ2*	*df*	CFI	Δ CFI	RMSEA	90% CI	Δ RMSEA
Tau-equivalent configural invariance	39.716	4	.996	–	.073	[.053, .094]	–
Tau-equivalent residual invariance	64.270	9	.994	−.002	.060	[.047, .074]	−.013

Means, standard deviations, percentages, skewness, kurtosis and correlations between loneliness and other measured variables are presented in Table 3. All correlations between TILS and criterion variables were as expected. The scales did not show extreme skewness nor kurtosis.

**Table 3:** Mean scores, standard deviations (*SD*), percentages and correlation coefficients between loneliness and criterion variables

Variable	Mean (*SD*) / Percentages	Skewness	Kurtosis	Loneliness
Loneliness	4.72 (1.71)	.88	−.11	–
Gender ^a^	56.1% females	–	–	−.01
Age	20.92 (2.65)	–	–	−.05**
General health	5.92 (2.10)	−.49	−.56	−.20**
Sleep quality	5.35 (2.15)	−.23	−.81	−.22**
Global self-esteem	5.97 (1.82)	−.65	.18	−.41**
Satisfaction with personal relationships	5.83 (2.40)	−.44	−.80	−.38**
Satisfaction with support received from friends	6.64 (1.87)	−.81	.40	−.37**
General quality of life	6.85 (1.40)	−.86	1.45	−.37**
Satisfaction with life	5.90 (1.88)	−.25	−.58	−.43**
Meaning in life	5.84 (2.08)	−.35	−.62	−.40**
Stress	10.75 (3.04)	.19	−.15	.41**
Anxiety	7.19 (4.55)	.63	−.08	.46**
Depression	4.64 (3.50)	.89	.38	.37**

^a^Point-biserial correlation coefficient (0 = female, 1 = male)

*p < .05. **p < .01

*Score ranges:* loneliness: 3–9, general health: 1–9, sleep quality: 1–9, global self-esteem: 1–9, satisfaction with personal relationships: 1–9, satisfaction with support received from friends 1–9, general quality of life: 1–9, satisfaction with life: 1–9, meaning in life: 1–9, stress: 4–20, anxiety: 0–21, depression: 0–21

## Discussion

The aim of the study was to address the problem of testing model fit in scales that consist of three items and to validate the Polish version of TILS in a student sample using the suggested psychometric approach. Factorial validity was supported by a good fit to the data of the tau-equivalent factorial model of TILS, and strict measurement invariance between genders using a very stringent tau-equivalent model. Cronbach’s alpha provided evidence for good reliability of the tool. The scale showed good concurrent validity. The results confirmed the expected correlations with the indicators of subjective well-being, perceived stress, anxiety, and depression, consistent with previous data showing worse psychosocial functioning of lonely individuals (Buelga et al., 2008; Cacioppo et al., 2006; Fanakidou et al., 2017; Heinrich & Gullone, 2006; Hughes et al., 2004; Liu & Guo, 2008; Matthews et al., 2017; Mellor et al., 2008; Moore & Schultz, 1983; Segrin & Passalacqua, 2010; Swami, et al., 2006; Tobiasz-Adamczyk et al., 2017; Yu et al., 2017). Lack of association with gender suggests adequate divergent validity. These, together with very good factorial structure and measurement invariance, support the construct validity of the scale. Therefore, the scale presents significant advantages for large scale epidemiological studies, expecially in the population of university students. Alongside other advancements in the measurement of loneliness (Auné, Abal, & Attorresi, 2020), including among the undergraduate students (Caballer et al., 2020), this study constitutes a significant contribution to the field.

Loneliness is an increasing problem among young generations (Cigna U.S. Loneliness Index, 2018; Twenge et al., 2019). More than 10% of the respondents in the current study answered that they often lack companionship, feel left out, or feel isolated from others. More than 56% of the students asnwered that some of the time or often they lack companionship, more than 43% feel left out, and 40% feel isolated from others. The correlations between loneliness and depression, anxiety, stress, self-esteem, general quality of life, satisfaction with life, and meaning in life ranged from .37 to .46 (absolute values). This means that there may be around 40% more students with considerably decreased well-being among lonely individuals (Rosenthal, 2005). With about 1.5 million students in Poland, and 10% being lonely, it would mean 60,000 students with anxiety and depression related to loneliness. To some extent it might explain such phenomena in Poland as the so called “crisis of child and adolescent psychiatry”, increasing suicide rates among underaged, and emotional and behavioral problems among affluent youth (see Atroszko & Atroszko, 2020). While these are crude and preliminary estimates, more studies on representative samples should analyze this problem in-depth, and provide more precise estimates.

### Strengths and Limitations

In terms of limitations, a convenience sample was used. Therefore, the results of the present study cannot be generalized to other populations without some reservations. The absence of a clinical evaluation of participants prevented any analyses and conclusions on the relationship between symptoms of disorders and results of the survey. Furthermore, all data were self-reported and are therefore prone to weaknesses of such data (such as social desirability bias, recall biases, common method bias, etc.). Metric and scalar invariance could not be tested due to the structure of the response scale (three-point response format). Instead, the configural model was compared directly to the strict model. This should be kept in mind if scales with more response options are tested. In such cases, metric and scalar invariance could be tested separately. Also, no other validated loneliness measures were used for testing the convergent validity of the instrument. A more detailed comparison of information from CFA and IRT/Rasch approaches has not been presented. Regarding the strengths of the present study, a large sample and valid psychometric tools were used. As far as the Authors are aware, it is the first practical application of a tau-equivalent model to testing factorial validity and measurement invariance of a three-item scale, and probably the first substantiated empirical case of tau-equivalent measurement invariance in psychological literature in general. This study has demonstrated how to overcome technical restrictions on psychometric testing in specific circumstances by applying the most rigorous assumptions, which can significantly improve the quality of research with ultra-short measures.

The aim of this study was not to present a new solution or “the solution” to the problem of factorial validity of three item scales. Instead, it analyses the existing solutions, discussing their advantags and disadvantages, and points to the optimal available option showing a relevant example of TILS. Such an analyses is clearly needed since none of the papers on the validity of the three item scales that the authors found in the relevant literature showed adequate factorial validity testing. The paper is mostly directed to non-experts in the psychometrics field for whom it may be an accesable description of a solution to the problem they may face in their research. Therefore, this paper constitutes a valuable and practical contribution to a wide range of fields applying ultra-brief psychometric measures.

### Conclusions and Future Studies Directions

This paper analysed the existing solutions to the problem of factorial validity of three item scales, discussing their advantags and disadvantages. Based on this analyses, an optimal available option is suggested along with a relevant example of TILS. Within CFA approach, tau-equivalent models allow for favorable psychometric performance of a scale, including better estimates of concurrent validity when a simple sum score is used instead of the latent score. However, it should be noted that when developing an ultra-short scale, different approaches could be applied and compared. Future studies could investigate the relative usefulness of using Rasch models or IRT models in comparison to CFA tau-equivalent models when creating a short scale. Perhaps more detailed information on the performance of particular items could allow for psychometrically better performing ultra-short scales and higher measurement invariance. Graded response model, a less restrictive approach than the Rasch model, could allow for items that contribute to the latent construct to varying degrees as it does not assume that all items have the same discrimination parameter. Since the tau-equivalent model is very restrictive, it could be recommended to use at least four-item scales, which allow testing the factorial structure of the more general congeneric model. Furthermore, uni- vs. multi-dimensionality of loneliness scales awaits more in-depth investigation, specifically more simulation studies in relation to developing ultra-short measures.

TILS showed good factorial validity, measurement invariance between genders, adequate concurrent and divergent validity, as well as good reliability in terms of internal consistency and test-retest measures. These results support the construct validity of the scale. Since loneliness seems to be a growing problem among younger populations, it is necessary to investigate its sources and consequences in more detail. Having a shorter tool for measuring loneliness can prove to be particularly useful in large surveys, which require the participants to fill in a sizable number of questionnaires, and when reducing the burden on the respondents is of great importance. Future research should look into its predictive validity in relation to health and well-being, particularly among young populations. More studies on loneliness among undergraduate students are highly warranted, as previous research indicates that this is a vulnerable population. In recent years indices of well-being among undergraduate and graduate students across countries tend to decline. The academic pressures that interfere with healthy social life leading to increasing feelings of loneliness, as well as social isolation, are suggested as one of the reasons. This has even more significance in the advent of global COVID-19 pandemic.

## Data Availability

The datasets generated during and/or analysed during the current study are not publicly available due to the fact that participants were not informed that the dataset will be openly available but are available from the corresponding author on reasonable request.
